# Dual Role of Transformer 2 Beta as Both a Developmental Necessity and a Disease Modulator

**DOI:** 10.3390/ijms26188805

**Published:** 2025-09-10

**Authors:** Evan Swarup, Hongyu Qiu

**Affiliations:** 1 Translational Cardiovascular Research Center, Department of Internal Medicine, College of Medicine-Phoenix, University of Arizona, Phoenix, AZ 85004, USA; eswarup@arizona.edu; 2 Clinical Translational Sciences (CTS) and Bio5 Institution, University of Arizona, Tucson, AZ 85721, USA

**Keywords:** transformer 2 beta, alternative splicing, RNA-binding protein, embryonic development, neurogenesis, neurological disorders, cancer

## Abstract

Transformer 2 beta (TRA2β) is a critical RNA-binding protein that regulates gene alternative splicing and is involved in cell cycle progression, neuronal differentiation, and cytoskeletal organization. It plays an essential role in embryonic development, particularly neurogenesis, where its deletion leads to severe cortical malformations and perinatal lethality. Dysregulation of TRA2β has been implicated in a range of diseases, including neurological, oncological, and immune-related disorders. Given its broad influence, TRA2β is a compelling candidate for targeted therapies and diagnostic biomarkers. This review highlights recent advances in our understanding of TRA2β regulation and its role in modulating alternative splicing across diverse cell types. It emphasizes TRA2β’s dual function as both a developmental regulator and a disease modulator and explores emerging insights into its therapeutic potential and future research directions. A deeper understanding of the cell-specific regulation of TRA2β may accelerate the development of innovative therapeutic strategies targeting this versatile protein.

## 1. Introduction

Alternative splicing (AS) is a fundamental biological process in which different combinations of exons from the same pre-mRNA are joined together, producing multiple distinct transcripts from a single gene. AS significantly increases the diversity of the proteome. Over 90% of human multi-exon genes undergo AS, underscoring their critical role in maintaining cellular and organismal homeostasis [1,2]. AS occurs in a tissue- and cell-specific manner, often resulting in major structural and, importantly, functional changes in protein. It often regulates gene expression through mechanisms such as inducing nonsense-mediated decay (NMD). NMD occurs when a premature stop codon is identified in an mRNA transcript. These mRNA are then targeted for degradation to prevent nonfunctional, truncated proteins from exhibiting deleterious effects on the cell. Premature stop codons may be identified during the first round of transcription or other rounds of NMD [3].

Exon inclusion is dependent on the splice site selection from the spliceosome. The spliceosome is a large molecular structure containing hundreds of different protein combinations referred to as small nuclear ribonucleoproteins (snRNPs) [4]. The spliceosome facilitates RNA–RNA, RNA–protein and protein–protein interactions in the translation process. The spliceosome is assembled through cis- and trans-acting factors. Cis-acting factors are specific exons and introns in pre-mRNA which serve as binding sites. Trans-acting elements are usually RNA binding proteins (RBPs) which bind to cis-acting factors in pre-mRNA. Different combinations of trans-acting and cis-acting elements work together to alter gene expression, impacting pre-mRNA splicing and polyadenylation, RNA stability, localization, RNA editing and translation [4]. One example of an RBP family is the tristetraprolin family, which binds to AU-rich elements in the 3′-untranslated regions, promoting removal of the polyA tail, subjecting mRNA to decay [5]. Another example is heterogeneous nuclear ribonucleoprotein (hnRNP) which assists in transforming newly formed heterogeneous nuclear RNAs to mRNAs, stabilizing mRNA during cellular transport and controlling mRNA translation through interaction with transcription factors [6].

One important RBP family is the transformer 2 (TRA2) family, which was first discovered in the fruit fly *Drosophila melanogaster* for its role in controlling sex-specificity [7,8,9]. Specifically, TRA2 was found to cooperate with the transformer (TRA) protein to regulate exon 4 inclusion in the double-sex gene included in females and excluded in males [7,9]. In humans, this family includes two homologues: transformer 2 alpha (TRA2α) and transformer 2 beta (TRA2β) [10,11,12]. TRA2α and TRA2β share approximately 75% identity, and 43% sequence homology with the Drosophila TRA2 protein [7]. The RNA recognition motif (RRM) domain, responsible for RNA binding, is highly conserved, being 85% identical between the two human TRA2α and TRA2β, and 54% identical to the Drosophila ortholog. TRA2α is believed to have evolved via duplication from TRA2β, indicating strong evolutionary conservation of this family [13].

Among the two, TRA2β is considered the more functionally significant member. The human TRA2β gene is located on chromosome 3q27.2 [14] and contains nine key exons encoding a protein with RRM and arginine/serine-rich (RS) domains. The RS domains facilitate interactions with other SR proteins and assist in RNA–RNA pairing, while the RRM domain specifically binds RNA, with a preference for the AGAA motif [15,16,17]. Emerging evidence has identified TRA2β as a key regulator of alternative splicing with essential roles in development and disease. Knockout studies have shown that TRA2β is indispensable for embryonic development, with complete loss resulting in embryonic lethality [18]. Beyond development, TRA2β is implicated in the pathogenesis of a variety of diseases, impacting multiple organ systems.

In this review, we summarize current advances in our understanding of TRA2β, focusing on its biological functions and the diversity of its isoforms. We highlight its roles in the development of germ cells, muscle tissue, and the nervous system. Furthermore, we examine recent research uncovering TRA2β’s involvement in disease processes, particularly neurological disorders, cancers, and immune system dysfunction. This growing body of knowledge offers valuable insights into TRA2β’s physiological and pathological relevance and lays the groundwork for future studies in both basic and translational biomedical research.

Articles were selected from PubMed using keywords such as “TRA2β”, “TRA2α”, “Transformer-2”, “RS domain”, and “RRM”. Journal articles were selected from dates ranging between 1993 and 2025 with more recently published literature from 2020 to 2025 being more extensively examined in this review.

## 2. TRA2β Protein Domains, Isoforms and Its Relationship to Family Members

**Figure 1:**
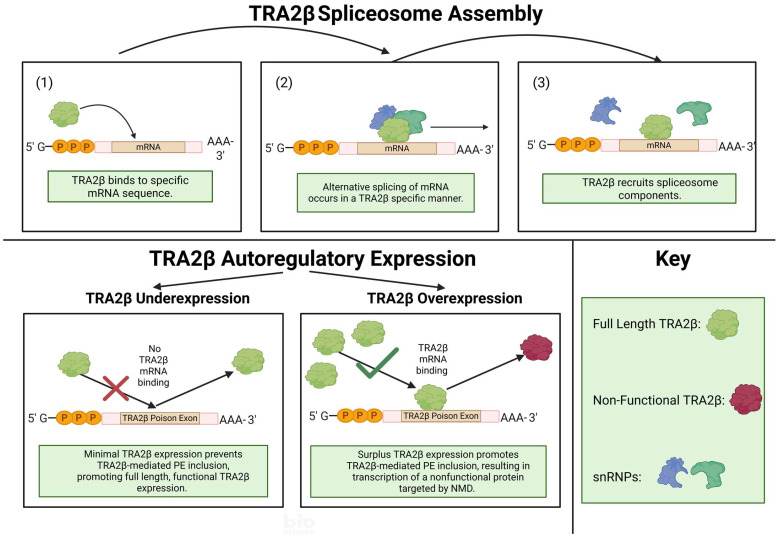
TRA2β spliceosome assembly and influence on AS of mRNA and TRA2β poison exon inclusion. TRA2β binds directly to cis factors on mRNA, providing trans-acting spliceosome components a location to assemble and facilitate AS. TRA2β also regulates its expression, directly binding to a poison exon in its sequence encoding nonfunctional proteins, which are a target for nonsense mediated decay. This figure was created using biorender.com.

Like other TRA2 family members, TRA2β protein contains an RRM flanked by two RS domains (RS1 and 2) on each side. These domains serve distinct roles. As elucidated in Figure 1, the RRM binds exon splicing enhancers and facilitates early spliceosome assembly by directly interacting with specific mRNA sequences. Exonic splicing enhancers are discrete sequences within exons which promote constitutive and regulated splicing [19]. The RS domains, located near the N- and C-termini, mediate protein–protein and protein–RNA interactions essential for splice site selection and exon inclusion [20,21]. RS domains may also help with the formation of RNA duplexes between U snRNAs and a branchpoint sequence. In vivo studies showed that RS domains are phosphorylated, facilitating RNA/protein activity [10]. TRA2β preferentially binds to the motif AGAA, a sequence frequently involved in alternative splicing [16,17]. Its RS domains promote interaction with other RS-containing proteins and support RNA–RNA base pairing. RRM carries out a specific function with mRNA [15,17].

**Figure 2:**
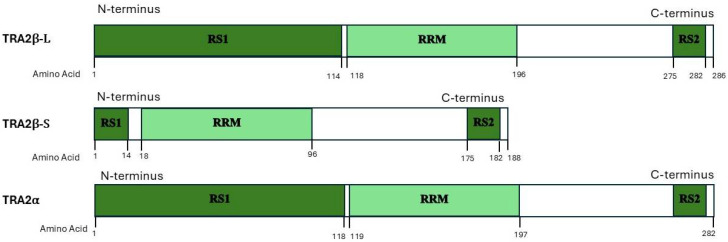
TRA2 significant isoforms. Detailed structure of TRA2β-L, TRA2β-S, and TRA2α, illustrating the general location of the arginine/serine rich regions 1 and 2 (RS1 and RS2) and the RNA recognition motif (RRM) in all three variants. Note that TRA2β-S has a truncated RS1 region in comparison to TRA2β-L.

As illustrated in Figure 2, TRA2β has different isoforms, primarily TRA2β-L and TRA2β-S. TRA2β-L encodes the full-length protein, which includes all nine exons in the sequence. This isoform includes the RS1 and RS2 domains as well as the RRM. TRA2β-S lacks exon 2 and instead initiates transcription within exon 3, producing a truncated protein. This isoform retains the RRM and RS2 domains but lacks the RS1 domain [17].

The two RS domains at different locations in TRA2 proteins serve different functions, and the RS1 domain contains more RS dipeptides than RS2. The difference in RS domain sizes is conserved across vertebrates and invertebrates, although the reason for this size difference in correlation to the function of each RS domain remains unknown [22]. In humans, RS1 domains are much more prominent in comparison to RS2, playing a pivotal role in splicing and regulation, while in Drosophila, the RS2 domain plays a more prominent role in female-specific gene transcription [10,22]. TRA2 proteins also contain a conserved tyrosine repeat near the C-terminus. The major divergence between the human and Drosophila homologs lies in the N-terminal RS domain, where human TRA2 proteins have 49 unique amino acids not found in Drosophila. This region represents the lowest sequence conservation [8,23].

Like other RS proteins, TRA2β contains a “poison exon”, a highly conserved, non-coding exon that introduces a premature stop codon when included. These poison exons are “ultra conserved” across species. The TRA2β poison exon, located between exons 1 and 2, is 100% identical between humans, mice, and rats, and 96% conserved across 300 million years of evolution in species such as chickens and lizards [24]. TRA2α also has a poison exon which is 306 nucleotides long. This poison exon is 48% conserved across vertebrates [22].

TRA2α and TRA2β show both redundancy and functional interplay. In MDA-MB-231 breast cancer cells, TRA2β binds to a poison exon in the TRA2α transcript, meaning that TRA2 upregulation can suppress TRA2α expression. However, only joint deletion of both TRA2α and TRA2β significantly impacts splicing of shared targets like CHEK1 exon 3. Mis-splicing of CHEK1 leads to elevated γH2AX, a marker of DNA damage, and reduced cell viability. These findings suggest TRA2 proteins play a vital role in maintaining genomic stability and may serve as therapeutic targets in cancer [16].

## 3. TRA2β in Embryogenesis and Systemic Development

TRA2β plays a critical role in the development of multiple organ systems, including germline, musculature, and nervous system. As an RNA-binding protein involved in alternative splicing, TRA2β regulates genes essential for embryogenesis, somitogenesis, and cell lineage specification, making it a pivotal factor in organismal development.

### 3.1. Role in Embryogenesis and Sex-Specificity

In mice, TRA2β plays an essential role in the early phases of embryo development. Widespread deletion of TRA2β in mice results in embryonic lethality around embryonic day 8 (E8), with affected embryos exhibiting severe growth retardation and a failure of organogenesis [18]. Despite its critical role in development, TRA2β appears functionally redundant in certain pathways, such as survival motor neuron 1 (SMN1) and SMN2 splicing, indicating a selective importance in specific splicing events [18].

The developmental lethality is likely tied to disrupted somitogenesis. In *Xenopus laevis* and *X. tropicalis*, TRA2β is broadly expressed during somitogenesis. Morpholino-oligonucleotide (MO) knockdown of TRA2β in *X. Laevis* or *X. tropicalis* resulted in delayed gastrulation, neural tube closure failure, inability to extend the anterior–posterior axis, and absence of mature somite formation. These defects are associated with widespread intron retention (RI) events in genes regulated by TRA2β. For example, in Xenopus, loss of TRA2β function results in RI inclusion in Wingless Integration Site 11B (WNT11b), a gene critical for somite formation. Specifically, inclusion of an alternative exon (exon 4) in WNT11b disrupts its expression and impairs mesodermal and somitic development, mirroring the morphological abnormalities seen in TRA2β-depleted embryos [25].

TRA2β’s ultra conserved poison exon plays an important role in spermatogenesis. In a conditional knockout model targeting this exon (TRA2β-cPEko) using CRISPR/CAS 9, male mice exhibited failed progression of germ cells past the pachytene stage of meiosis, leading to widespread apoptosis. This disruption results in molecular defects in germline cells and dysregulated expression of key meiotic genes

TRA2β expression also directly regulates poison exon inclusion. When overexpressed, TRA2β binds to the poison exon in its own mRNA, promoting inclusion, making TRA2β’ a target for NMD. This process is essential to regulate male gametogenesis, and also explains why the exon is so highly expressed across vertebrate genomes [24].

Together, these findings highlight TRA2β as a master regulator of developmentally important alternative splicing events, essential for embryonic survival, somitogenesis, and germ cell maturation. Its tightly regulated expression and splicing, particularly through its poison exon, underscores its importance in ensuring precise temporal and spatial gene regulation during development.

### 3.2. Muscle Development

In addition to the development of embryos and germ cells, TRA2β is also critical for muscle differentiation and developmental splicing decisions across smooth and striated muscle lineages.

TRA2β regulates myogenesis by modulating alternative splicing of muscle-related transcripts. In MO knockdown models, 10 out of 11 key myogenic genes were significantly downregulated, including caveolin-1 (cav1) and myosin heavy chain 4 (myh4). These data suggest that TRA2 is required for the proper splicing and expression of genes involved in muscle fiber formation and maturation during development [25].

It has been shown that TRA2β governs the splicing of myosin phosphatase target subunit 1 (Mypt 1), a key determinant of smooth muscle contractility phenotype, resulting in the developmental switch between phasic and tonic muscle contraction in mice and rats [26]. Between post-natal day (PND) 14 and 56, a developmental switch leads to Mypt1 exon 24 inclusion (E24^+^), driving a shift from tonic (slow) to phasic (fast) smooth muscle contraction in tissues such as the mesenteric artery, portal vein, and bladder. Conditional, smooth muscle-specific knockout of TRA2β (using Smmhc-CreERT2) impairs E24 inclusion and prevents this contractile phenotype switch. Animal models also confirm TRA2β’s role in this process by demonstrating tissue-specific regulation of smooth muscle splicing and contractile properties [26].

TRA2β-modulated Mypt1 splicing alters sensitivity to nitric oxide (NO) and cGMP-dependent relaxation, especially in phasic muscles such as the portal vein and mesenteric artery. Loss of TRA2β shifts Mypt1 splicing to the E24^−^ (LZ^+^) isoform, enhancing responsiveness to cGMP signaling, and thus illustrating TRA2β’s specific tuning of smooth muscle physiology [27].

Distinct isoforms of the TRA2β, namely TRA2β-L and TRA2β-S, have divergent roles in skeletal muscle differentiation. In chicken myogenic cells, TRA2β-L was generally stable throughout embryonic muscle development and differentiation, while TRA2β-S was significantly upregulated. In addition, overexpression of TRA2β-L inhibited myotube formation and myoblast fusion, while the opposite occurred for TRA2β-S overexpression. Despite their contradictory functions, the two isoforms share similar RNA targets. These targets mainly function in the cell cycle and RNA metabolism [10,13,28]. Combined deletion of both isoforms with siRNA resulted in decreased cell proliferation and expression of important myogenic markers: myoblast determination protein 1 (MyoD1), myogen (MyoG), and myosin heavy chain (MyHC).

Additionally, TRA2β targets include transforming growth factor beta receptor 2 (TGFBR2), a negative regulator of myogenesis. TRA2β influences alternative splicing of TGFBR2 exons 1 and 2, potentially modulating TGF-β pathway activity during muscle differentiation. Upon binding with these TGF-B ligands, TGFBR2 phosphorylates TGFBR, which activates mothers against decapentaplegic homologues 2 and 3, acting as a gatekeeper for TGF-B signaling, inhibiting myogenesis. While preliminary research has been performed on the impacts of TRA2β on TGFBR2 splicing, further research needs to be conducted to examine the impacts of TRA2β on specific TGFBR2 isoforms [13].

Together, these findings position TRA2β as a versatile regulator of skeletal and vascular muscle development through context-dependent isoform and splicing control.

### 3.3. Neurological Development

One reason TRA2β KO results in embryonic lethality [18] is due to its critical role in the development of the brain. Compared to other tissues, the brain exhibits a higher frequency of AS events. Dysregulation of a single AS protein can disrupt the tightly regulated processes essential for neurogenesis. This is proven in mouse models, where the conditional knock out (cKO) of TRA2β results in perinatal lethality, with pups surviving only up to approximately 36 h after birth, indicating that TRA2β plays a vital role in early neural development [29]. Therefore, differential alternative splicing of TRA2β influences the development of structural systems within the brain, and its dysregulation may contribute to neurodegenerative disease development and scattered neuronal structures.

#### 3.3.1. Cerebral Cortex Development

The cerebral cortex, the outermost layer of gray matter in the brain tissue, is responsible for higher-level cognitive functions such as memory, thinking, learning, reasoning, problem-solving, emotion regulation, and sensory processing [30]. These complex tasks require a diverse array of cells, including projection neurons, interneurons, and glia. The development of this area in the brain begins in the ventricular zone, where progenitor cells develop various types of cortical projection neurons, which eventually split into different regions of the cerebral cortex [31,32].

In mice, TRA2β is broadly expressed throughout the brain by embryonic day 5 (E5) with significant upregulation in the developing cerebral cortex [29]. Specifically, from E11.5 to postnatal day 0 (P0), TRA2β is highly expressed in neural progenitors, cortical projection neurons, and radial glial cells. It is also present in ventricular and subventricular zones from 14.5 days to 16.5 days post-coitum [21,29]. Conditional knock out (CKo) of TRA2β in mice using EMX1-Cre results in missing cerebral cortex and hippocampus structures as well as ventriculomegaly, or significant enlargement of the ventricles of the brain [21,29]. Additionally, TRA2β cKO mice lack approximately 85% of their neocortex, illustrating the significance of TRA2β in the development of brain tissue [33].

Widespread apoptosis of neural progenitors in the TRA2β KO mice occurred at E11.5, coinciding with peak TRA2β expression, indicating a direct role of alternative splicing in the projection and proliferation of cortical neurons during neurogenesis. Despite these anatomical defects, no gross behavioral differences were detected in mice with specific TRA2β cKO. However, dysregulation of splicing in genes like microtubule- associated protein tau (Mapt) can lead to neurodegeneration and dementia [21].

On the molecular level, TRA2β expression influences the inclusion of several key exons, including exon 4 in Shugoshin-like protein 2 (Sgol2) and exon 4 in Tubulin Delta 1 (TUBD1). Shugoshin proteins (encoded by Sgol2) ensure proper sister chromatid cohesion during meiosis I, while TUBD1 helps translocate and elongate the nucleus during spermatogenesis. RA2B also regulates the splicing of exon 5 in clathrin light chain B (Cltb) and exon 10 in Mapt, which are involved in processes such as fertility, spermatogenesis, endocytosis, microtubule stability, and cell cycle progression, respectively. Additionally, TRA2β directly influences the expression of p21 and tNASP, a testis- and embryo-specific isoform of the nuclear autoantigenic sperm protein, which plays an important role in the progression of the cell cycle. P21 is an inhibitor of the cell cycle through inhibiting cyclin-dependent kinases 2 and 4 (CDK2/CDK4), resulting in G1 cell cycle arrest. In neuronal precursor cells (NSC34) development, the absence of TRA2β leads to significant upregulation of p21 and downregulation of tNASP, resulting in increased apoptosis, as observed in TRA2β mouse brains [29].

#### 3.3.2. The Visual System

Cortical-specific TRA2β cKO also impacts the development of the dorsal lateral geniculate nucleus (dLGN). The dLGN is part of a “circuit” consisting of the retinal ganglion cells and the cortex. The dLGN and ventral lateral geniculate nucleus (vLGN) are crucial for relaying information from the retina to the brain. Cortical tissue impacts the development of the dLGN system in juvenile monkeys and cats, helping facilitate the connection of neurons within the “circuit” and the cortex [33].

At E18.5, TRA2β cKO restricts axon branching in the dLGN but not the vLGN. Despite slight apoptosis of dLGN neurons, the gross morphology remains generally spared. However, in vivo electrode analysis through light response of the dLGN revealed that dLGN neurons fail to transmit light-evoked signals, suggesting impaired retinal projection due to TRA2β cKO-mediated apoptosis. In addition, TRA2β cKO mice perform poorly in an orientation selectivity test, illustrating a functional impairment of the visual cortex. This confirms that TRA2β is essential for developing a fully functional visual system, largely through its influence on cortical development and axonal connectivity [33].

## 4. Contribution to the Pathogenesis of Various Diseases

In addition to its essential role in development, emerging evidence has shown that TRA2β is also widely involved in various diseases, particularly neurological disorders, cancer, and immune system dysfunctions. Recent advances in related research are discussed below and also summarized in Table 1.

### 4.1. Neurological Disease

While TRA2β plays an extremely vital role in the development of brain tissue, it remains essential in managing neural hemostasis after birth. Dysregulation of TRA2β in vivo has been shown to lead to several neurodevelopmental disorders and neurodegenerative diseases in humans and animal models.

Neurodevelopmental Disorders: Neural function relies heavily on proper mRNA alternative splicing due to its central role in neuronal gene regulation. Improper AS of neurological genes may result in neurodevelopmental disorders, impacting brain development and function [14].

A notable but unnamed neurodevelopmental disorder is marked by an aberrant increase in a truncated TRA2β isoform known as TRA2β-S. Patients with elevated TRA2β-S expression exhibit symptoms including developmental delay, y epilepsy, behavioral abnormalities, feeding disorders, growth retardation, and visual abnormalities [17]. In a study of lymphoblastoid and fibroblast cells from developmentally delayed human brains, decreased expression of TRA2β-L and increased expression of TRA2β-S were also found. In HEK 293 cells, both TRA2β-L and TRA2β-S competitively bind to normal TRA2 binding sites, such as CHEK1. While TRA2β-1 normally promotes inclusion of exon 3 in CHEK1, overexpression of TRA2β-3 (via GFP-tagged vector) led to exon 3 exclusion, likely due to the truncated RS1 domain. This competitive binding impairs splicing regulation and may underlie some phenotypes of neurodevelopmental disorders [17].

Another clinical case involving a patient presenting with seizures at five months postnatally showed elevated levels of TRA2β-3 truncated isoform. The patient also experienced similar symptoms to the previous study, such as skeletal abnormalities, hypotonia, spasms, and nonspecific facial abnormalities. However, the patient did not experience behavioral abnormalities, possibly due to her young age [14].

Alzheimer’s Disease (AD): TRA2β also plays a role in the progression of AD, a neurodegenerative disease mainly marked by the accumulation of amyloid-β (Aβ) plaque in the brain [47,48]. Aβ toxicity is partly mediated through interactions with the receptor for advanced glycation end-products (RAGE), which exists in two isoforms: membrane-bound mRAGE (full transmembrane protein) and soluble secretory esRAGE, which contains partial RAGE transcript but serves as a decoy receptor [49].

In postmortem human AD brains, a decreased TRA2β expression is associated with an elevated ratio of mRAGE and esRAGE [34]. In the specific SCI-RNA KO model, TRA2β suppression significantly reduced levels of esRAGE and increased levels of mRAGE [34]. This suggests TRA2β plays a role in regulating the alternative splicing of RAGE. The elevated mRAGE levels and reduced esRAGE (which acts as a decoy receptor for Aβ) in the context of TRA2β suppression likely enhances the interaction between Aβ and RAGE. This, in turn, contributes to the advancement of AD pathogenesis. Additionally, decreased glucose metabolism, commonly seen in AD, was shown to further reduce TRA2β levels, exacerbating disease progression [34].

Interestingly, TRA2β and another splicing factor, hnRNP A1 (heterogeneous nuclear ribonucleoprotein A1), have opposite effects on RAGE splicing in the AD model. hnRNP A1 is known to bind to similar splice sites as TRA2β does but induce opposing effects. While TRA2β promotes esRAGE production, hnRNP A1 encourages mRAGE splicing. These competing interactions may mirror similar dynamics seen in diseases like spinal muscular atrophy [34].

Spinal Muscular Atrophy (SMA): SMA is a debilitating, autosomal neurodegenerative disease characterized by the loss of lower motor neurons, resulting in the atrophy of muscles. At the molecular level, SMA is caused by insufficient expression of survival motor neuron (SMN). In humans, there are two SMN gene paralogs, SM1 and SM2, which differ by only five nucleotides [50]. A transition from C to T in the sixth nucleotide of exon 7 in SMN2 disrupts creates a novel hnRNP A1-binding splicing silencer, leading to exon 7 skipping, encoding a dysfunctional protein resulting in SMA pathogenesis [51].

In the SM2 gene, an AG-rich sequence (GAARGARR) is recognized by TRA2β. In vitro, TRA2β-1 promotes exon 7 inclusion in mouse testis [35]. Consequently, TRA2β has been investigated as a potential therapeutic target. One current therapeutic mechanism considered is antisense oligonucleotide (ASO), which either binds to target mRNA sites and prevents translation, or uses ASOs that have long RNA tails which translation machinery recognizes and transcribes instead of the target sequence [52].

However, conflicting in vitro and in vivo data limit its clinical potential. For instance, in vitro heat treatment of SMA promoted SMN2 exon 7 inclusion through a TRA2-mediated mechanism [36] suggesting the therapeutic potential of TRA2β in treating SMA. However, in vivo studies show that only extremely high levels of TRA2β had an impact on SMN2 exon 7 inclusion in mice [36]. Additionally, cKO of TRA2β in motor neurons did not produce any major SMA-like phenotype in mice [36]. These findings indicate that other splicing factors may play a more important role in SMN2 splicing in vivo.

Interestingly, in vivo heat treatments of mouse cells with SMA, survival motor neuron 2 (SMN2) exon 7 inclusion is promoted and modulated through TRA2β upregulation, suggesting that thermal regulation may be a promising therapeutic avenue [36].

Moreover, small molecules like valproic acid (VPA) and M344 are known to upregulate TRA2β expression [53]. VPA, used for conditions like bipolar disorder, migraine, epilepsy, and spinal muscular atrophy with anti-cancer side effects, also promotes splicing inclusion of SM2 exon 7 via TRA2β mediated mechanism [29].

### 4.2. Cancers

TRA2β has been widely studied in cancer biology, with evidence supporting its role in the progression of multiple cancer types, including breast, cervical, ovarian, and colon cancer, and squamous cell carcinoma [41]. TRA2β expression usually promotes cancer metastasis and is usually lethal in these instances [38]. In many of these contexts, TRA2β functions as a proto-oncogene, influencing AS of genes involved in cell proliferation, adhesion, and survival. Its expression often correlates with poor prognosis and high metastatic potential [38].

Previous studies have correlated high TRA2β expression to increased transcription factor and estrogen levels. Specifically, high TRA2β expression potentially stem from HSF1 and ETS-1 transcription factor expression. TRA2β expression may also be controlled by estrogen, in which estrogen is a receptor for positive breast cancer cell development [54].

While its oncogenic role has been reviewed previously [10], recent research continues to explore TRA2β’s specific therapeutic potential in ovarian cancer [23], squamous cell carcinoma [38,39,40], and colon adenocarcinoma [41]. Here we highlight these recent progressions that go beyond the previous review.

Ovarian Cancer: Ovarian cancer is an aggressive gynecological cancer often associated with physical deformity due to invasive treatments. TRA2β has been shown to promote the progression of ovarian cancer in humans [37]. TRA2β downregulates cell adhesion genes, indicating an impact on the extracellular matrix. TRA2β also upregulates cell cycle genes such as nuclear autoantigenic sperm protein (NASP) [37,55], mothers against decapentaplegic homolog 4 (SMAD4), DSN1 component of MIS12 kinetochore complex (DSN1), mini chromosome maintenance 8 (MCM8), and ubiquitin conjugating enzyme E2 (UBE2I) [23]. It also enhances mitotic gene expression. TRA2β upregulation was directly correlated with increased malignancy of ovarian cancer, reinforcing its role as a proto-oncogene in this disease [23].

Squamous Cell Carcinoma (SCC): SCC originates in mucosal epithelial cells and is usually caused by exposure to carcinogens such as opium, tobacco, or alcohol consumption. SCC is prevalent in oral, head, neck, and lung cancers and has a relatively low 5-year survival rate of 40–50% [39].

TRA2β has been implicated in the function of several SCCs. For example, TRA2β is upregulated in human oral SCC (OSCC) [39]. Specific knockout of TRA2β results in decreased OSCC cell proliferation and increased apoptosis [39]. TRA2β activates the glycogen synthase kinase 3-β-catenin (GSK3/β-catenin) pathway leading to the destabilization of β-catenin, a protein associated with cancer progression signaling pathways, increasing the splicing of proliferation and differentiation genes such as Meis homeobox 1 (MEIS1) and programmed cell death 6 (PDCD6) [39]. TRA2β knockout also decreased cyclin D1 and c-Myc expression, further elucidating its role in promoting oncogenic processes in OSCC [39].

Additionally, the cBioPortal clinical database shows that TRA2β was upregulated in 20% of head and neck SCC (HNSCC) and 47.7% in lung SCC (LUSCC) patients. TRA2β also interacts with other mRNA splicing proteins which play key roles in the carcinogenic process, such as RNA-binding motif protein-X (RBMX), heterogeneous nuclear ribonucleoprotein C (HNRNPC), and heterogeneous nuclear ribonucleoprotein H1 (HNRNPH1).

There are contradictory findings across SCC subtypes. For example, in HNSCC, patients with higher levels of TRA2β survived longer than patients with lower TRA2β levels. However, in LUSCC, there was no significant difference in patient survival and TRA2β expression [40]. These findings contradict other data found on SCC of the cervix, which has been linked to lower human patient mortality [38]. Therefore, further studies need to be performed with TRA2β and SCC to properly characterize the relationship.

Adenocarcinoma and Epithelial Cancer: Epithelial–mesenchymal transition (EMT) is a key mechanism in cancer metastasis, where cells lose their epithelial traits and acquire attributes of mesenchymal cells. AS produces different proteins that orchestrate the transformation from epithelial to mesenchymal cells during this process. For example, serpine1 is the most enriched mRNA in RNA-induced silencing complex (RISC) during this process, which is enriched in colon adenocarcinoma [41]. Serpine1 has similar splicing target sites to TRA2β. In colon adenocarcinoma, serpine1 suppresses TRA2β, interfering with miRNA regulation and downregulates CD8+ T cells in tumors, promoting tumorigenesis [41].

While TRA2β, in general, is the more significant paralogue in the RS protein family, in some circumstances, TRA2α plays a significant role. Cell lines of pancreatic epithelial adenocarcinoma (PANC0504), epithelial lung adenocarcinoma (NCI-H23), and brain epithelial adenocarcinoma (LN319) have higher expression of TRA2α than TRA2β [42]. This suggests that, in some cell lines, TRA2α can compensate for the lesser expression of TRA2β. While specific KO of either TRA2α or TRA2β causes minimal changes in splicing, joint knockdown of both paralogues resulted in 4.5–7-fold greater changes in skipping specific target exons [42]. In TRA2α-dependent cells, CRISPR-Cas9-mediated TRA2B cKO increased the amount of cell death significantly [42].

Glioblastoma: Glioblastoma is representative of 48.3% of primary brain tumors [56]. This type of cancer is extremely malignant, with a median survival of 14.6 months post-diagnosis and usually only 5.5% of patients survive past diagnosis [57]. TRA2B downregulation aligns with decreased cancer metastasis [44]. In glioblastoma cell lines, CRISPR-CAS9-mediated deletion of homeotic2-like protein (ASH2L), a transcriptional regulator, leads to less TRA2β expression, along with glioblastoma cell apoptosis, and cell cycle arrest, through reducing cell cycle genes, such as TRA2β [44]. ASH2L could serve as a potential mechanism to reduce cancer cell metastasis. TRA2B’s implications in glioblastoma again highlight the therapeutic potential this protein has in the realm of cancer therapeutics [44].

Acute Myeloid Leukemia: Acute myeloid leukemia is an aggressive malignancy which stems from immaturely transformed myeloid cells. AML cells have extremely high proliferative capacity and antiapoptotic activity [58]. Myelopoiesis, the development of myeloid cells (e.g., monocytes, dendritic cells, macrophages), is regulated in part by alternative splicing mechanisms involving Toll-like receptors (TLRs). PRPF40A, a pre-mRNA processing factor involved in splicing, shares limited overlap in targets with TRA2β, but impacts immune regulation. High PRPF40A expression has been linked to reduced AML progression, though its relationship with TRA2β in this context remains under investigation [43].

### 4.3. Immune System Disorders

TRA2β contains an ultra-conserved poison exon, which is an approximately 200 base pair long sequence. Inclusion of the poison exon results in NMD of TRA2β, thereby preventing functional TRA2β protein production [59]. These poison exons serve as regulatory elements to fine-tune protein levels via splicing control.

In mouse CD8+ T cells from C57BL/6-Tg (TcraTcrb)1100Mjb/Crl (OT-I) mice, TRA2β poison exon splicing triggers T cell differentiation from naive to effector cells. Poison exon inclusion results in smaller T cells with less surface area and significantly lower levels of interferon gamma (IFN-γ) and tumor necrosis factor alpha (TNFα) secretion and production [45]. When the poison exon is included, TRA2β reduces the expression of signaling lymphocytic activation molecule family 6 (SLAMF6) due to decreased inclusion of exon 7. Poison exon inclusion also dictates antigen sensing and suppresses both T cell receptor (TCR) and JAK-STAT signaling. These observations demonstrate that TRA2β’s autoregulatory poison exon is critical for proper immune modulation and effector function of T cells through alternative splicing [45].

NMD, a post-transcriptional quality control mechanism that degrades mRNAs containing premature stop codons, accounts for 20% of monogenic diseases. Drug treatments often modulate nonsense-mediated decay efficiency, inducing single nucleotide polymorphisms (SNPs) post-transcriptionally into the sequence, which may result in NMD. TRA2β, due to its poison exon, is itself a direct target of NMD. Harringtonine (HHT), a drug used in the treatment of myeloid leukemia, has been shown to inhibit NMD in a dose-dependent manner, which may stabilize TRA2β transcript [60].

Rheumatoid arthritis (RA) is an autoimmune disorder characterized by joint inflammation and degradation of the synovial membrane. RA progression involves numerous immune cells releasing cytokines, upregulating chronic inflammation pathways. Necroptosis is a programmed form of cell death that has inflammatory properties, often leading to extracellular matrix damage, cartilage destruction, and pain. TRA2β has been proposed to play a role in promoting necroptosis in RA, although the precise molecular mechanism remains unclear and warrants further investigation [46].

HIV is a retrovirus that integrates into the host genome and produces approximately 40 distinct mRNA transcripts, driving viral replication and virion production. Because the virus relies on alternative splicing, SR proteins like TRA2β are pivotal to HIV gene expression. Overexpressing TRA2β reduced expression of critical HIV structural proteins GAG and ENV. This effect is accompanied by changes in viral mRNA splicing, although the viral protein essential for nuclear export of HIV RNA is not directly affected by TRA2β [20]. However, different splice variants play different roles in HIV pathogenesis, specifically being that the N-terminal RS domain is very highly active in HIV modulation while the C-terminal domain is not. Both terminals bind to the same spots on TRA2β RNA but have different impacts on pathogenesis. Increased TRA2β expression resulted in less HIV mRNA, indicating its repressive role in viral replication [20]. Interestingly, TRA2B exerts a more potent effect on HIV splicing regulation than its paralogue TRA2α, further highlighting its significance in the context of viral immune evasion [20].

## 5. Therapeutic Potential and Future Perspectives

TRA2β is a central regulator of alternative splicing with critical roles in development, disease pathogenesis, and immune function. Its broad biological impact makes it a compelling candidate for targeted therapies and diagnostic biomarkers in neurodevelopmental, neurodegenerative, oncological, and immunological diseases.

Type 2 diabetes mellitus (T2DM) is a disease characterized by hyperglycemia and insulin resistance. Glucagon-like peptide-1 (GLP-1) is a hormone that alleviates insulin resistance. Importantly, GLP-1 levels influence TRA2β expression, suggesting a link between alternative splicing regulation by TRA2β and sortilin isoform production in metabolic disease [34].

One current TRA2B pharmacological therapeutic is antisense oligonucleotides (ASOs). ASOs are short, synthetic single stranded oligodeoxynucleotides which help alter protein expression at the RNA level [61]. ASOs function based on Watson–Crick base recognition and are therefore able to be flexibility designed [62].

A TRA2β/ASO mediated therapeutic mechanism has been briefly studied in cancer. Overexpression of TRA2β is associated with less survival across several types of cancer, including breast cancer, cervical SCC, acute myeloid leukemia (AML), lung adenocarcinoma, and cutaneous melanoma. In these cases, the decreased inclusion of the TRA2β-PE isoform correlates with poor outcomes. ASO-1570, an antisense oligonucleotide, promotes TRA2β-PE exon inclusion, triggering NMD of TRA2β transcripts. ASO-1570-induced downregulation of TRA2β shows anti-cancer effects in glioblastoma, triple-negative breast cancer, and colorectal cancer, making ASO-1570 a potential therapeutic target [38].

Antisense TRA2β splice-modulating therapies, such as ASOs that promote poison exon inclusion, offer a promising strategy to reduce TRA2β activity in cancers. However, current ASO-mediated therapeutic mechanisms face significant limitations, as ASOs often accumulate in vital organs like the liver and spleen, exhibiting cytotoxic effects, necessitating research on drug delivery mechanisms. Potential off-target binding and self-hybridization is also a major concern in the ASO drug discovery related field [62]. Therefore, ASO-specific treatment illustrates the therapeutic potential of TRA2β, but requires in vivo validation and clinical trials to establish safety and efficiency.

Therapeutic mechanisms targeting TRA2β need to be examined further. TRA2β plays an important role in modulating T cell activation and antigen sensing [45]. TRA2β could become a novel target for immune checkpoint modulation or vaccine enhancement, especially in cancer and chronic infections.

Despite these therapeutic potentials, the full therapy targeting TRA2β remains largely untapped. In the future, TRA2β research should aim to elucidate tissue-specific mechanisms. Understanding how TRA2β regulates splicing in different tissues, especially in the brain, immune system, and tumors, will clarify its context-dependent roles in health and disease. Additionally, for validation of TRA2β biomarkers, TRA2β expression patterns and isoform profiles may serve as biomarkers for early detection, prognosis, or treatment response in diseases such as AD, SMA, and various cancers. Comprehensive understanding of TRA2’s biomarkers in conjunction with tissue-specific mechanisms will fully unlock this important protein’s therapeutic potential.

Discrepancies between in vitro and in vivo data, especially in diseases like SMA, underscore the need for more physiologically relevant models to accurately assess TRA2β’s therapeutic utility.

As our understanding of RNA biology expands, TRA2β stands out as a key regulatory node with broad implications for developmental biology, neurology, oncology, and immunology. Targeting its splicing activity holds considerable promise for precision medicine.

## 6. Conclusions

**Figure 3:**
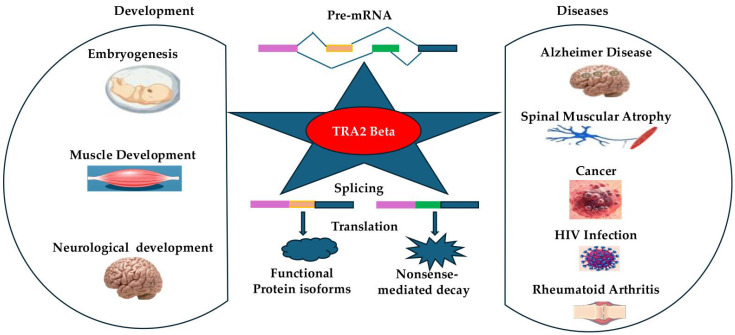
The dual role of TRA2β in development and diseases. TRA2β controls the splicing of genes involved in cell cycle progression, cytoskeletal organization, and neuronal differentiation, and plays an essential role in embryogenesis, muscle and neurological development. In adults, dysregulation of TRA2β also contributes to a range of diseases, including neurological, oncological, and immune-related disorders, through regulating relevant gene splicing. Color coding of the bar represents the different exons.

As summarized in Figure 3 TRA2β is a critical RNA-binding protein involved in alternative splicing regulation across multiple biological systems. It plays an essential role in embryonic development, particularly in neurogenesis, where its deletion results in severe cortical malformations and perinatal lethality. TRA2β regulates the splicing of genes involved in cell cycle progression, neuronal differentiation, and cytoskeletal dynamics, highlighting its importance in brain structure and function.

Beyond development, TRA2β dysregulation contributes to various diseases. In neurological disorders, aberrant isoforms of TRA2β have been linked to intellectual disability, epilepsy, and AD, via altered splicing of genes like CHEK1 and RAGE. In spinal muscular atrophy, TRA2β promotes the inclusion of SMN2 exon 7. TRA2β also acts as a proto-oncogene in multiple cancers, including ovarian, squamous cell, and glioblastoma, where it promotes tumor growth and metastasis through splicing regulation of cell cycle and adhesion genes. Additionally, TRA2β modulates immune responses by controlling T cell differentiation and cytokine production and has been implicated in diseases such as HIV and rheumatoid arthritis.

Despite its critical role in developmental biology and disease, therapies directly targeting TRA2β remain a clinical gap and a significant challenge. A deeper understanding of its cell-specific functions and regulation of alternative splicing will advance our knowledge and accelerate the discovery of targeted therapeutic strategies.

## Figures and Tables

**Table 1:** The contribution of TRA2β to the various diseases discussed in this review.

Disease/Disorder	Organism/Cell Type	Relevant Mechanism	References
Neurodevelopmental Disease	Human-derived lymphoblastoid cells analyzed ex vivo.	Increased TRA2β-S and decreased TRA2β-L disrupts CHEK1 exon 3, resulting in developmental delay, epilepsy, seizures, and facial abnormalities.	[14,17]
Alzheimer’s Disease (AD)	Human neuroblastoma SH-SY5Y cells studied in vitro.	TRA2β reduces full-length mRAGE but increases truncated esRAGE, preventing Aβ-RAGE interaction, reducing AD pathogenesis.	[34]
Spinal Muscular Atrophy (SMA)	Mouse testis cell lines, GC-1 spermatogenia, SMN2 −/−; SMN2 +/+ spinal neuron cells and cortical neurons studied in vitro.	TRA2β promotes SMN2 exon 7 inclusion in vitro but shows limited in vivo effects unless highly overexpressed.	[35,36]
Ovarian Cancer (OC)	Human cervical carcinoma cell line HeLa (CCTCC CDC0009) studied in vitro.	TRA2β promotes progression by upregulating cell cycle/mitotic, and downregulates cell adhesion.	[23,37]
Squamous Cell Carcinoma (SCC)	Human MDA-MB231, T84, U87-MG, A375, NCI-H647, SK-OV-3, and 5637 cell lines studied in vitro. Additionally, human head and neck and lung squamous cell carcinoma from the Cancer Genome Atlas database studied in vivo	Upregulated TRA2β in SCC promotes tumor growth via splicing and proliferation and survival genes.	[38,39,40]
Adenocarcinoma	Normal mouse mammary gland epithelial cells and HEK293T, A549, LN229, and LN319 cell lines studied in vitro.	TRA2β influences splicing and tumor progression. It is suppressed by Serpine1.	[41,42]
Acute Myeloid Leukemia	Human HL-60 cells studied in vitro.	Competitively binds with PRPF40A, a known acute myeloid leukemia inhibitor. Relationship needs further characterization	[43]
Glioblastoma	U87MG, U373, and T98G human cell lines studied in vitro.	Reduced TRA2β leads to apoptosis and cell cycle arrest, reduced glioblastoma progression,	[44]
T Cell Differentiation	Mouse splenic OT-I cells in vivo/in vitro	TRA2β poison exon inclusion triggers T cell differentiation.	[45]
Rheumatoid Arthritis	Human rheumatoid arthritis patient immune cell from GEO database.	TRA2β promotes necroptosis and inflammation.	[46]
HIV Infection	HEK 293T cells transfected with pAdML dsx or pHxb R-/RII plasmids studied in vitro.	TRA2β suppresses HIV replication, reducing GAG and ENV protein levels.	[20]

## Data Availability

Not applicable.
